# Knowledge, Attitude, and COVID-19 Vaccination Uptake Among Drug Dispensers in Community Pharmacies: A Cross-Sectional Study

**DOI:** 10.24248/eahrj.v9i1.820

**Published:** 2025-09-30

**Authors:** Baraka Fundo, Betty Maganda, Deogratias Katabalo, Stanley Mwita

**Affiliations:** a Department of Pharmaceutics and Pharmacy Practice, Catholic University of Health and Allied Sciences, Mwanza, Tanzania; b Department of Pharmaceutics and Pharmacy Practice, Muhimbili University of Health and Allied Sciences, Dar es Salaam, Tanzania.

## Abstract

**Background:**

COVID-19 is a respiratory disease caused by Severe Acute Respiratory Syndrome coronavirus 2 (SARS-COV-2). Drug dispensers are at risk of contracting COVID-19. This study aimed to assess the knowledge, attitude, and COVID-19 vaccination uptake among drug dispensers in community pharmacies.

**Methods:**

A cross-sectional study was conducted among drug dispensers in community pharmacies in Dar es Salaam from January to June 2022. Participants in the study were pharmacists, pharmaceutical technicians, pharmaceutical assistants, and Accredited Drugs Dispensing Outlets (ADDO) dispensers. The calculated minimum sample size was 360. A stratified sampling technique was used to obtain the number of pharmacies and drug dispensers required for each district. Data was collected using a self-administered, semi-structured questionnaire. To determine the predictors of COVID-19 vaccination status by the drug dispensers, multivariable binary logistic regression was performed.

**Results:**

A total of 375 participants were included. More than half (57.0%) of participants had good knowledge of COVID-19 vaccines, while 203 (54.1%) of respondents had a positive attitude. About 111 (29.6%) of respondents had received the COVID-19 vaccine. Drug dispensers aged 50 years and above and pharmacists were more likely to have received the COVID-19 vaccine (AOR 58.82; 95% CI, 7.407 to 500.0; *P*<.001) and (AOR 5.025; 95% CI, 2.062 to 12.346, *P*=.005), respectively.

**Conclusion:**

Drug dispensers in community pharmacies possessed a good level of knowledge regarding COVID-19 vaccination; however, gaps in attitude and vaccine uptake persisted. Strengthening educational campaigns through professional associations, utilizing digital platforms for information dissemination, and integrating vaccine promotion into continuing professional development programs can enhance their role in public health response efforts.

## BACKGROUND

COVID-19 is a respiratory disease caused by Severe Acute Respiratory Syndrome Coronavirus-2 (SARS-COV-2).^1^ The novel human coronavirus disease 2019 (COVID-19) was reported in Wuhan, China, in 2019.^2^ By July 20, 2022, there were more than 570 million confirmed cases and over 6.4 million lives lost to the disease.^3^ SARS-CoV-2 is transmitted between humans through direct contact, aerosol droplets, the faecal-oral route, and intermediate fomites from both symptomatic and asymptomatic patients during the incubation period.^4^

Several control measures were instituted by various countries around the world as recommended by the World Health Organization (WHO) to prevent the transmission of the COVID-19 pandemic.^5^ The most promising strategy to confine the pandemic and provide hope to reduce mortality and morbidity rates depends on the use of effective, safe, and affordable antiviral agents and vaccines.^6^ There are no specific antiviral therapies for COVID-19, and vaccination against COVID-19 is considered one of the most cost-effective health interventions to prevent and control the pandemic. However, to be effective, a vaccine must be accepted and used by a large majority of the population.^7^

Tanzania, like other countries, has also experienced the pandemic. Up to early July 2022, there had been 37,510 confirmed cases of COVID-19, with 841 deaths reported.^8^ The Tanzanian government has been equally implementing the WHO-recommended measures to prevent COVID-19. Mass vaccinations against the pandemic were introduced in the country in August 2021 in 550 centers set up by the government.^9^ To date, there are more than 1,548 vaccination centers in the country. So far, vaccines approved for immunization in Tanzania include Johnson & Johnson's Janssen COVID-19, Pfizer-BioNTech COVID-19, Sinopharm (BIBP COVID-19), Moderna-mRNA, and Sinovac.^10^ Despite the Tanzanian government's sincere efforts to ensure massive vaccination and an increase in awareness, the number of people, including healthcare workers, vaccinated is still low. As of February 2023, a total of 32,093,549 people had been fully vaccinated in the United Republic of Tanzania^11^, which translates to about 45% of the total population. This number is lower than the estimated 70 to 85% of the population that needs to be vaccinated to reach the herd immunity threshold.^12^ Various studies have shown that reasons for COVID-19 vaccine hesitancy are safety concerns, confusion over protection levels, perceived risk and fears, poor health literacy, conspiracy theories aimed against vaccination, lack of awareness about the virus, misinformation or lack of accurate knowledge about the vaccines, and concerns about safety in the elderly and people with various pre-existing comorbidities.^13–17^ The COVID-19 pandemic and its associated vaccine have highlighted vaccine hesitancy among healthcare workers. Reasons for hesitancy include concerns about safety and efficacy, mistrust of government and institutions, waiting for more data, and feeling that personal rights are being infringed upon.^18^

In resource-limited countries such as Tanzania, pharmaceutical drug outlets are often patients’ first point of contact with the healthcare system and the preferred channels for purchasing medicine. Community drug dispensers working in pharmacies that sell pharmaceutical drugs serve as primary healthcare providers.^19^ Community dispensers are more frequently in contact with healthcare seekers than any other healthcare professionals. Thus, they are a vulnerable group to be infected with COVID-19. Community drug dispensers can play a vital role in increasing immunization awareness among their clients who access services in drug pharmacies if they are well-informed and have accepted the vaccines. In addition, unvaccinated community drug dispensers pose a risk of transmitting COVID-19 to their clients. The identification of factors associated with vaccination status is necessary to inform policy changes and help public health experts develop a conceptual framework and educational campaign aimed at increasing awareness in the general population.

Although global efforts have been made to promote COVID-19 vaccination, hesitancy remains a significant public health concern, particularly among healthcare providers who are expected to serve as role models and sources of credible information. In Tanzania, limited data exist on the knowledge, attitudes, and vaccination status of frontline pharmacy personnel, particularly drug dispensers in community pharmacies, who interact daily with the public and are key sources of health information. Existing national surveys have focused on broader populations such as the general public or hospital-based healthcare workers, with little attention paid to this unique cadre. This study aimed to fill that gap by assessing the knowledge, attitude, and COVID-19 vaccination status of drug dispensers in Dar es Salaam, Tanzania, thereby informing targeted public health interventions, policy formulation, and the development of a conceptual framework for vaccine advocacy in the community pharmacy setting.

## MATERIALS AND METHODS

### Study design and population

A cross-sectional study was conducted among drug dispensers in community pharmacies in the country's commercial capital, Dar es Salaam. Data were collected between January and June 2022, which corresponds to a period after the initiation of the national vaccination campaign in August 2021 and during ongoing efforts to expand coverage. Participants in the study were pharmacists, pharmaceutical technicians, pharmaceutical assistants, and Accredited Drugs Dispensing Outlets (ADDO) dispensers. Students in practical training were excluded from the study. The minimum sample size was 360. It was calculated by using a prevalence of 62.5%^20^ as a good knowledge rate in Leslie Kish's formula for cross-sectional studies. A stratified sampling technique was used to obtain the number of pharmacies and drug dispensers required for each district. The sampling was stratified based on the five administrative districts of Dar es Salaam: Ilala, Kinondoni, Temeke, Ubungo, and Kigamboni. The number of pharmacies and drug dispensers selected from each district was proportionate to the total number of registered community pharmacies in that district, using data provided by the Pharmacy Council of Tanzania. Within each stratum, simple random sampling was applied to select individual participants.

### Data Collection

Data was collected using a self-administered, semi-structured questionnaire. The questionnaire was validated by two experts from the departments of epidemiology and pharmacy practice. It was adopted and modified based on the literature.^12,20–23^ The questionnaire was available both online as a Google survey form and physically. The knowledge and attitude COVID-19 vaccine questionnaire had internal consistency, with Cronbach's alpha values of 0.740 and 0.721, respectively. To reduce the potential bias introduced by self-reported data, participants were assured of the confidentiality and privacy of their responses. A pilot sample (n = 12) was used to improve the wording and clarity of expression of the survey items. Data from the pilot sample was not used in any further analysis. Based on the pilot results, minor modifications were made to the wording and structure. The final version of the questionnaire required an estimated time of 5–10 minutes to complete. The questionnaire was developed and distributed in English. The information collected included sociodemographic data of the participants, knowledge, attitude, and vaccination status regarding COVID-19 vaccines.

The questionnaire consisted of 10 knowledge questions. Each correct or yes response scored 1 point; others scored 0. Total general vaccine knowledge subscale scores ranged from 0 to 10. A score of 7–10 was regarded as good knowledge, while 6–4 and 0–3 were regarded as moderate and low, respectively. The participants’ attitudes towards COVID-19 vaccines were scored using seven items on a three-point Likert scale. For positive questions, the scoring was agreed = 3, neutral = 2, and disagree = 1, while for negative questions, the scoring system was vice versa. A score between 15 and 21 was deemed indicative of a positive attitude, while a score between 1 and 14 indicated a negative attitude.^12,22,23^

### Ethical considerations

This study was approved by the Muhimbili University of Health and Allied Sciences (MUHAS) institutional review board with reference number DA.25/111/01B/187. Permissions were also obtained from relevant authorities, and informed consent was sought from study participants. Confidentiality and privacy were strictly maintained during the data collection process.

### Data Analysis

The data collected via the Google Survey form was extracted into Microsoft Excel and subsequently integrated with the data obtained from hard-copy questionnaires. The final data was then exported to IBM SPSS Statistics for Windows version 26.0 (IBM Corp, Armonk, NY, USA) for analysis. Data were presented as percentages and tables. To determine the predictors of COVID-19 vaccination status by the drug dispensers, multivariable binary logistic regression was performed. The adjusted odds ratio (AOR) values and their 95% confidence intervals (95% CI) were calculated. A *P* value of less than .05 was considered statistically significant.

## RESULTS

### Sociodemographic Information

This study recruited 375 participants; the response rate was 96.2%. The majority of participants were female (54.7%). The median (interquartile range (IQR)) age was 30 (21–68). Most of the participants were young adults <40 years old, married, and pharmaceutical technicians, accounting for 78.7%, 64.3%, and 54.1%, respectively. Detailed demographics are presented in Table 1.

**TABLE 1:** Sociodemographic Characteristics of the Participants (N=375)

Characteristics	n	%
Age group		
20–29	178	47.5
30–39	117	31.2
40–49	57	15.2
≥50	23	6.1
Median (IQR)	30 (21–68)	–
Sex		
Male	170	45.3
Female	205	54.7
Marital status		
Single	134	35.7
Married	241	64.3
Profession		
Pharmaceutical technician	203	54.1
Pharmaceutical assistant	39	10.4
ADDO dispenser	47	12.5
Pharmacist	86	22.9

### COVID-19 Experience

About 45.1% of participants had experienced COVID-19 symptoms. The result indicated that 61.9% of participants had family members who had ever experienced COVID-19 symptoms. In this study, 73.5% of participants reported having ever attended to clients at their pharmacies due to COVID-19-related symptoms. About 26.9% reported having lost family members due to COVID-19, and about 30.2% had lost their clients due to COVID-19.

### Knowledge of COVID-19 Vaccines

More than half (57.0%) of participants showed good knowledge of COVID-19 vaccines, while one-quarter (25.0%) had a moderate knowledge score, and 18.0% of respondents had low knowledge. Respondents were asked 10 questions to assess their general knowledge of COVID-19 and COVID-19 vaccines, and their answers are shown in Table 2. The majority of respondents knew that vaccines are important for protection against infectious diseases (97.3%), COVID-19 vaccines are effective (66.1%), authorized COVID-19 vaccines are safe (63.5%), and COVID-19 vaccines have some side effects or adverse events (78.7%). Most of the participants were also able to mention examples of COVID-19 vaccines that have been authorized to be used in Tanzania (79.7%). The study also found that the main source of information about COVID-19 vaccines is the internet and social media (64.9%).

**TABLE 2:** Responses on Knowledge and Sources of Information about COVID-19 Vaccines

S/N	Variables	Responses	
		n	%
1	Do you think vaccines are important for protection against infectious diseases?		
	Yes	365	97.3
	No	4	1.1
	Not sure	6	1.6
2	What examples of vaccines do you know?		
	Correct	355	94.7
	Wrong	20	5.3
3	How do vaccines work?		
	Correct	333	88.8
	Wrong	42	11.2
4	Do you know anything about COVID-19 vaccines?		
	Yes	292	77.9
	No	18	4.8
	Not sure	65	17.3
5	Do COVID-19 vaccines help in controlling the disease?		
	Yes	238	63.5
	No	34	9.1
	Not sure	103	27.4
6	Do you understand the effectiveness of COVID-19 vaccines?		
	Yes	248	66.1
	No	30	8.0
	Not sure	97	25.9
7	Is it safe to receive an authorized COVID-19 vaccine?		
	Yes	238	63.5
	No	34	9.1
	Not sure	103	27.4
8	Mention some examples of COVID-19 vaccines that have been authorized to be used in Tanzania.		
	Correct	299	79.7
	Wrong	76	20.3
9	Are there any side effects or adverse events related to COVID-19 vaccination?		
	Yes	295	78.7
	No	6	1.6
	Not sure	74	19.7
10	Which population group do you think is not eligible for vaccination?		
	Correct	88	23.5
	Wrong	287	76.5
11	The main sources of information		
	Mainstream media	74	19.7
	Internet/social media	243	64.9
	Friends/colleagues	49	13.1
	Training/Education	9	2.3

### Attitude Towards COVID-19 Vaccines

Results revealed that 203 (54.1%) of respondents had a positive attitude toward COVID-19 vaccines, while 172 (45.9%) exhibited a negative attitude. The majority of 232 (61.9%) disagreed that COVID-19 vaccines have elemental substances that can harm their health. About 217 (58.9%) agreed that drug dispensers and other healthcare providers should be given priority for vaccines, while 216 (57.6%) agreed that COVID-19 vaccines are important for people of old age. (Table 3).

**TABLE 3:** Attitude Towards Covid-19 Vaccines

S/N	Variables	Agree n (%)	Neutral n (%)	Disagree n (%)
1	COVID-19 disease is a conspiracy from developed nations	41 (10.9)	159 (42.4)	175 (46.7)
2	COVID-19 vaccines are meant for business	43 (11.5)	138 (36.8)	194 (51.7)
3	COVID-19 vaccines have elemental substances that can harm our health	23 (6.1)	120 (32.0)	232 (61.9)
4	COVID-19 vaccines do not help to reduce the spread, effects and deaths related with COVID-19 disease	49 (13.1)	102 (27.2)	224 (59.7)
5	COVID-19 vaccines are important for people with old age	216 (57.6)	113 (30.1)	46 (12.3)
6	COVID-19 vaccines can alter our genetic makeup	37 (9.9)	110 (29.3)	228 (60.8)
7	Drug dispensers and other healthcare providers should be given priority for vaccine	217 (57.9)	116 (30.9)	42 (11.2)

### Uptake and Predictors of the COVID-19 Vaccination

In the current study, 111 (29.6%) of respondents had received the COVID-19 vaccine, while 264 (70.4%) were not vaccinated. The multivariable binary logistic regression revealed that age, marital status, and profession were significantly associated with vaccination status. Drug dispensers aged 50 and above were more likely to have received the COVID-19 vaccine compared to those aged between 20 and 29 years (AOR 58.82; 95% CI, 7.407 to 500.0, *P*<.001). The married participants were more likely to have been vaccinated compared to singles (AOR 2.444; 95% CI, 1.406 to 4.237; *P*=.002). Further, pharmacists were more likely to have received the COVID-19 vaccine compared to pharmaceutical technicians (AOR 5.025, 95% CI, 2.062 to 12.346, *P*=.005). (Table 4).

**TABLE 4:** Predictors of Vaccination Status for Covid-19 Vaccine

	AOR	95%CI	*P Value*
Age group			
20–29	Ref		
30–39	1.572	0.900–2.732	.108
40–49	2.820	1.477–5.405	.002
>50	58.82	7.407–500.00	< .001
Sex			
Male	Ref		
Female	0.494	0.205–1.193	.117
Marital status			
Single	Ref		
Married	2.444	1.406–4.237	.002
Profession			
Pharmaceutical Technician	Ref		
Pharmaceutical Assistant	0.387	0.048–3.115	.372
ADDO dispenser	0.007	0.005–0.012	.998
Pharmacist	5.025	2.062–12.346	.005

## DISCUSSION

This study aimed to assess the knowledge, attitude, and COVID-19 vaccination status of drug dispensers in Dar es Salaam, Tanzania. COVID-19 is a highly contagious worldwide epidemic that affects people. Because they are on the frontline of the fight against this highly contagious disease and have direct contact with infected individuals, healthcare personnel are more likely to contract the disease than the general population.^24^ Thus, healthcare providers need to understand preventive measures and have a positive attitude toward them. About 45.1% of the respondents in this study had ever experienced at least one COVID-19 symptom. The reported prevalence is higher compared to the findings from previous studies (12.9%-38.2%).^25,26^ The difference could be attributed to the differences in study settings and the period of data collection. In agreement with a previous study,^27^ about three-quarters of the respondents had ever been visited by clients with complaints of COVID-19 symptoms. This could be explained by the fact that pharmaceutical drug pharmacies are often patients’ first point of contact with the health care system and preferred channels for purchasing medicines.

The current study revealed that 57% of drug dispensers had good knowledge about the COVID-19 vaccination. This finding is consistent with another study that was conducted among healthcare workers in Dubai.^28^ On the contrary, other studies done among healthcare workers in China,^29^ and Ethiopia,^20^ reported higher levels of COVID-19 vaccination knowledge, i.e., 89.2% and 62.5%, respectively. The possible reason for the inconsistency might be differences in the study population; previous studies included all healthcare workers.

The majority of respondents in this study knew that COVID-19 vaccines are effective and that COVID-19 vaccination has some side effects or adverse events. However, they lacked knowledge about the recommended COVID-19 vaccines in Tanzania. Compared to the findings of this study, previous research reported higher rates of knowledge among healthcare workers regarding COVID-19 vaccine effectiveness (95.9%) and adverse events (92.9%), and lower rates of recommended COVID-19 vaccines (87.7%).^30^

This study also found that the main source of information about COVID-19 vaccines is the internet and social media. The finding is in line with the reports from previous studies conducted in Palestine,^22^ and Jordan,^23^ but it is not in agreement with the other study conducted in Ethiopia,^31^ that reported the mainstream media to be the main source of information. These findings highlight the importance of the internet, social media, and mainstream media such as TV and radio in the dissemination of information related to COVID-19.

Almost half (54.1%) of the participants in this study reported having a positive attitude towards COVID-19 vaccines. This finding is in line with the previous study that was conducted among healthcare workers (52.3%),^20^ and contrary to the findings from the general population (44.7%).^12^ The diversity in attitude between healthcare workers and the general population could be attributed to healthcare workers’ higher exposure to the virus, understanding of the vaccine's benefits, and trust in scientific data and medical guidelines. The majority of the participants in this study disagreed that COVID-19 vaccines have elemental substances that can harm their health and agreed that drug dispensers and other healthcare providers should be given priority for vaccines and that COVID-19 vaccines are important for people of older age. The previous study conducted in India revealed that the majority of participants agreed that they feel fully protected from COVID-19 infections in the future after getting the COVID-19 vaccine (68.8%) and that although the COVID-19 vaccine appears to be safe, there may be problems that we haven't yet discovered (42.2%). ^32^

Less than one-third (29.6%) of the participants in this study reported having received the COVID-19 vaccine. The proportion of drug dispensers who were vaccinated in this study was low compared to the reports from the study conducted in China (34.9%),^29^ among healthcare workers and higher than the study done in Sudan by Yassin et al. (22.7%).^33^ The low rate of vaccination among healthcare workers is attributed to hesitancy due to concerns about the safety and efficacy of the COVID-19 vaccine, mistrust of authorities and pharmaceutical companies, and waiting for more data from clinical trials.^18,34^ In this study, drug dispensers aged 50 and above, married, and pharmacists were reported to be more likely to receive the COVID-19 vaccine. Older healthcare workers, especially those aged 50 and married, tend to have more experience and understanding of the benefits of vaccination, increasing their likelihood of being vaccinated. Marital status often corresponds with family responsibilities, motivating married individuals to protect their loved ones. Pharmacists have higher education levels and greater access to accurate vaccine information, enhancing their vaccine acceptance. The previous study that was conducted in Turkey reported that advanced age, male gender, living with family, having a child, having a chronic disease, and having a high fear of COVID-19 were the factors affecting the willingness to accept the COVID-19 vaccine.^35^ To enhance COVID-19 vaccine uptake among community drug dispensers, it is recommended that targeted educational campaigns be developed and implemented through professional associations and regulatory bodies, ensuring the messaging is trusted and profession-specific. Leveraging digital platforms for the dissemination of accurate, evidence-based information can counteract misinformation and broaden outreach. Furthermore, integrating vaccine advocacy and education into continuing professional development (CPD) programs will reinforce the importance of immunization as part of professional practice. Lastly, empowering and actively involving community pharmacies in public health initiatives—such as vaccination campaigns—can amplify their role as accessible and influential healthcare providers within the community.

### Limitations of the Study

There are several limitations to the study. First, the use of a cross-sectional survey method means that causal inferences cannot be drawn. Second, there's a chance that respondents overreported their vaccination status and positive attitudes due to social desirability bias, which occurs when people answer questions in a way that they think others would find favorable. Third, the study was conducted in one region only, thus limiting the results’ national generalizability. Lastly, factors such as personal health status, religious beliefs, political views, and access to healthcare services—which could influence vaccination decisions—were not measured in this study.

## CONCLUSION

Drug dispensers in community pharmacies possess a generally good level of knowledge regarding COVID-19 vaccination; however, gaps in attitude and vaccine uptake persist. Strengthening educational campaigns through professional associations, utilizing digital platforms for information dissemination, and integrating vaccine promotion into continuing professional development programs can enhance their role in public health response efforts. Future research should employ qualitative methods to explore the specific reasons, perceived barriers, and motivational factors behind COVID-19 vaccine hesitancy among different categories of drug dispensers.

## References

[1] Gan L, Chen Y, Hu P, Wu D, Zhu Y, Tan J, Li Y, Zhang D. Willingness to receive SARS-CoV-2 vaccination and associated factors among Chinese adults: a cross sectional survey. International journal of environmental research and public health. 2021;18(4):1993. 10.3390/ijerph18041993 33670821 PMC7922368

[2] Gong F, Xiong Y, Xiao J, Lin L, Liu X, Wang D, et al. China's local governments are combating COVID-19 with unprecedented responses—from a Wenzhou governance perspective. Frontiers of Medicine. 2020 Apr;14:220–4. 10.1007/s11684-020-0755-z 32166600 PMC7089477

[3] World Health Organization. Living guidance for clinical management of COVID-19. 2021. Accessed: April 23, 2024. https://www.who.int/publications/i/item/WHO-2019-nCoV-clinical-2021-2

[4] Huang C, Wang Y, Li X, Ren L, Zhao J, Hu Y, et al. Clinical features of patients infected with 2019 novel coronavirus in Wuhan, China. The lancet. 2020 Feb 15;395(10223):497–506. 10.1016/S0140-6736(20)30183-5 PMC7159299 31986264

[5] Nicola M, Alsafi Z, Sohrabi C, Kerwan A, Al-Jabir A, Iosifidis C, et al. The socio-economic implications of the coronavirus pandemic (COVID-19): A review. International journal of surgery. 2020 Jun 1;78:185–93. 10.1016/j.ijsu.2020.04.018 32305533 PMC7162753

[6] Fiolet T, Kherabi Y, MacDonald CJ, Ghosn J, Peiffer-Smadja N. Comparing COVID-19 vaccines for their characteristics, efficacy and effectiveness against SARS-CoV-2 and variants of concern: a narrative review. Clinical Microbiology and Infection. 2022 Feb 1;28(2):202–21. 10.1016/j.cmi.2021.10.005 34715347 PMC8548286

[7] Yang J, Liao Y, Hua Q, Lv H. A survey of awareness of COVID-19 knowledge, willingness and influencing factors of COVID-19 vaccination. Vaccines. 2022 Mar 28;10(4):524. 10.3390/vaccines10040524 35455273 PMC9027136

[8] World Health Organization. WHO COVID-19 dashboard, The United Republic of Tanzania Situation 2021. Accessed: June 5, 2024. https://covid19.who.int/region/afro/country/tz

[9] United Nations International Children's Emergency Fund. First COVID-19 vaccines have arrived in Tanzania. 2021. Accessed: August 5, 2024. https://www.unicef.org/tanzania/press-releases/first-covid-19-vaccines-have-arrived-tanzania

[10] Tanzania Medicines and Medical Devices Authority (TMDA). Frequently asked questions about COVID-19 vaccines. 2021. Accessed: July 15, 2024. https://www.tmda.go.tz/uploads/medialibrary/1625138184-FAQs-on-COVID-19-Vaccines-1.pdf

[11] From below 10 to 51 percent - Tanzania increases COVID-19 Vaccination Coverage. Accessed: July 15, 2024. https://www.afro.who.int/countries/united-republic-of-tanzania/news/below-10-51-percent-tanzania-increases-covid-19-vaccination-coverage

[12] Abebe H, Shitu S, Mose A. Understanding of COVID-19 vaccine knowledge, attitude, acceptance, and determinates of COVID-19 vaccine acceptance among adult population in Ethiopia. Infection and drug resistance. 2021 Jun 1: 2015–25. 10.2147/IDR.S312116 PMC8179743 34103948

[13] Steinert JI, Sternberg H, Prince H, Fasolo B, Galizzi MM, Büthe T, et al. COVID-19 vaccine hesitancy in eight European countries: Prevalence, determinants, and heterogeneity. Science advances. 2022 Apr 27;8(17):eabm9825. 10.1126/sciadv.abm9825 35476432 PMC9045608

[14] Elhadi M, Alsoufi A, Alhadi A, Hmeida A, Alshareea E, Dokali M, et al. Knowledge, attitude, and acceptance of healthcare workers and the public regarding the COVID-19 vaccine: a cross-sectional study. BMC public health. 2021 May 20;21(1):955. 10.1186/s12889-021-10987-3 34016073 PMC8136114

[15] Nasr L, Saleh N, Hleyhel M, El-Outa A, Noujeim Z. Acceptance of COVID-19 vaccination and its determinants among Lebanese dentists: A cross-sectional study. BMC Oral Health. 2021 Dec;21:1–0. 10.1186/s12903-021-01950-0 34587930 PMC8479009

[16] Issanov A, Akhmetzhanova Z, Riethmacher D, Aljofan M. Knowledge, attitude, and practice toward COVID-19 vaccination in Kazakhstan: a cross-sectional study. Human vaccines & immunotherapeutics. 2021 Oct 3;17(10):3394–400. 10.1080/21645515.2021.1925053 34044728 PMC8437534

[17] Cordina M, Lauri MA. Attitudes towards COVID-19 vaccination, vaccine hesitancy and intention to take the vaccine. Pharmacy Practice (Granada). 2021 Mar;19(1). 10.18549/PharmPract.2021.1.2317 PMC8005329 33828623

[18] Peterson CJ, Lee B, Nugent K. COVID-19 vaccination hesitancy among healthcare workers—a review. Vaccines. 2022 Jun 15;10(6):948. 10.3390/vaccines10060948 35746556 PMC9227837

[19] Tsuyuki RT, Beahm NP, Okada H, Al Hamarneh YN. Pharmacists as accessible primary health care providers: Review of the evidence. Can Pharm J. 2018;151(1):4–5. 10.1177/1715163517745517 PMC5755826 29317929

[20] Adane M, Ademas A, Kloos H. Knowledge, attitudes, and perceptions of COVID-19 vaccine and refusal to receive COVID-19 vaccine among healthcare workers in northeastern Ethiopia. BMC public health. 2022 Jan 18;22(1):128. 10.1186/s12889-022-12543-z 35042476 PMC8765812

[21] Abay ES, Belew MD, Ketsela BS, Mengistu EE, Getachew LS, Teferi YA, Zerihun AB. Assessment of attitude towards COVID-19 vaccine and associated factors among clinical practitioners in Ethiopia: A cross-sectional study. PloS one. 2022 Jun 16;17(6):e0269923. 10.1371/journal.pone.0269923 35709076 PMC9202929

[22] Al-Kafarna M, Matar SG, Almadhoon HW, Almaghary BK, Zaazouee MS, Elrashedy AA, et al. Public knowledge, attitude, and acceptance toward COVID-19 vaccines in Palestine: a cross-sectional study. BMC Public Health. 2022 Mar 17;22(1):529. 10.1186/s12889-022-12932-4 35300647 PMC8930193

[23] Lataifeh L, Al-Ani A, Lataifeh I, Ammar K, AlOmary A, Al-Hammouri F, et al. Knowledge, attitudes, and practices of healthcare workers in Jordan towards the COVID-19 vaccination. Vaccines. 2022 Feb 9;10(2):263. 10.3390/vaccines10020263 35214721 PMC8875156

[24] Ehrlich H, McKenney M, Elkbuli A. Protecting our healthcare workers during the COVID-19 pandemic. The American journal of emergency medicine. 2020 Jul;38(7):1527. 10.1016/j.ajem.2020.04.024 32336585 PMC7162741

[25] Cabas P, Di Bella S, Giuffre M, Rizzo M, Trombetta C, Luzzati R, Antonello RM, Parenzan K, Liguori G. Community pharmacists’ exposure to COVID-19. Research in Social and Administrative Pharmacy. 2021 Jan 1;17(1):1882–7. 10.1016/j.sapharm.2020.06.003 32499159 PMC7255346

[26] Khojah HM, Itani R, Mukattash TL, Karout S, Jaffal F, Abu-Farha R. Exposure of community pharmacists to COVID-19: a multinational cross-sectional study. Journal of Taibah University Medical Sciences. 2021 Dec 1;16(6):920–8. 10.1016/j.jtumed.2021.07.004 34305508 PMC8282887

[27] Zaidi ST, Hasan SS. Personal protective practices and pharmacy services delivery by community pharmacists during COVID-19 pandemic: results from a national survey. Research in Social and Administrative Pharmacy. 2021 Jan 1;17(1):1832–7. 10.1016/j.sapharm.2020.06.017 33317761 PMC7347346

[28] Albahri AH, Alnaqbi SA, Alnaqbi SA, Alshaali AO, COVID-19 among healthcare workers in primary healthcare centers in Dubai: a cross-sectional survey, 2020. Frontiers in Public Health. 2021 Jul 28;9:617679. 10.3389/fpubh.2021.617679 34395350 PMC8355417

[29] Li XH, Chen L, Pan QN, Liu J, Zhang X, Yi JJ, et al. Vaccination status, acceptance, and knowledge toward a COVID-19 vaccine among healthcare workers: a cross-sectional survey in China. Human Vaccines & Immunotherapeutics. 2021 Nov 2;17(11):4065–73. 10.1080/21645515.2021.1957415 34344260 PMC8828068

[30] Hong J, Xu XW, Yang J, Zheng J, Dai SM, Zhou J, et al. Knowledge about, attitude and acceptance towards, and predictors of intention to receive the COVID-19 vaccine among cancer patients in Eastern China: A cross-sectional survey. Journal of integrative medicine. 2022 Jan 1;20(1):34–44. 10.1016/j.joim.2021.11.002 34774463 PMC8559872

[31] Tesfaye ZT, Yismaw MB, Negash Z, Ayele AG. COVID-19-related knowledge, attitude and practice among hospital and community pharmacists in Addis Ababa, Ethiopia. Integrated Pharmacy Research and Practice. 2020 Aug 24: 105–12. 10.2147/IPRP.S265226 PMC7455591 32904494

[32] Dara S, Sharma SK, Kumar A, Goel AD, Jain V, Sharma MC, et al. Awareness, attitude, and acceptability of healthcare workers about COVID-19 vaccination in Western India. Cureus. 2021 Sep;13(9). 10.7759/cureus.17796 PMC8556728 34729277

[33] Yassin EO, Faroug HA, Ishaq ZB, Mustafa MM, Idris MM, Widatallah SE, Abd El-Raheem GO, Suliman MY. COVID-19 vaccination acceptance among healthcare staff in Sudan, 2021. Journal of Immunology Research. 2022;2022(1):3392667. 10.1155/2022/3392667 35155687 PMC8832156

[34] Holzmann-Littig C, Braunisch MC, Kranke P, Popp M, Seeber C, Fichtner F, Littig B, et al. COVID-19 vaccination acceptance and hesitancy among healthcare workers in Germany. Vaccines. 2021 Jul 12;9(7):777. 10.3390/vaccines9070777 34358193 PMC8310090

[35] Kaplan AK, Sahin MK, Parildar H, Adadan Guvenc I. The willingness to accept the COVID-19 vaccine and affecting factors among healthcare professionals: A cross-sectional study in Turkey. International Journal of Clinical Practice. 2021 Jul;75(7):e14226. 10.1111/ijcp.14226 33864328 PMC8250279

